# Root canal morphology of native Tanzanian permanent mandibular molar teeth

**DOI:** 10.11604/pamj.2018.31.24.14416

**Published:** 2018-09-12

**Authors:** Habiba Suleiman Madjapa, Irene Kida Minja

**Affiliations:** 1 Dental Department, Muhimbili National Hospital, Dar es Salaam, Tanzania; 2 Department of Restorative Dentistry, Muhimbili University of Health and Allied Sciences, Dar es Salaam, Tanzania

**Keywords:** Mandibular molar, morphology, root canal, configuration, Tanzanian

## Abstract

**Introduction:**

Research has shown variations in morphology of root canals to differ amongst ethnic groups. We aimed to investigate the root morphology and canal configuration of permanent mandibular molars in a native Tanzanian population.

**Methods:**

146 first and 85 second mandibular molars were collected from Tanzanian patients. After removal of the pulp tissues and staining using Methylene blue ink from the canal system, the teeth were decalcified and rendered clear using 98% methyl salicylate. The teeth were then examined under magnification of 10X for: number of roots, tooth length, number of canals, location of apical foramen, presence of an apical delta and canal configuration using Vertucci's classification.

**Results:**

All mandibular molars had two separate roots. The mean tooth length for mandibular 1^st^ and 2^nd^molars were 21.7 mm and 20.5mm, respectively, with no statistically significant difference in mean tooth length between males and females. All the mesial roots 1st and 2^nd^ mandibular molars possessed two root canals, while 40.4% and 54.1% of the distal roots of 1^st^ and 2^nd^ molars, respectively, had two canals. The majority of the examined teeth had their apical foramen located centrally, with an apical delta present in the distal root of one-second molar. Root canal configuration types commonly reported were Type II in the mesial and Type I in the distal roots of the mandibular 1^st^ molar; while the 2^nd^ molar had, respectively, root types II / IV and type I.

**Conclusion:**

There were observed variations in the morphology of root canals in a Tanzanian population. Caution is advised to clinicians when performing root canal treatment.

## Introduction

Research has shown variations in morphology of root canals to differ amongst ethnic groups [1]. This discovery instigated more studies that were conducted amongst various ethnic populations to determine the frequency of these occurrences of anatomical variation in the root canal morphology of their populations [2, 3]. Adequate knowledge of the root canal morphology and its common variations in a population will assist clinicians in their plan to perform root canal treatment. The number of root canals, the presence of lateral canals, accessory canals, the location of apical foramen, presence of an apical delta, and root canal configuration type are important variants in root canal morphology that have a direct impact on the success of the root canal treatment (RCT). These variations usually lead to failure due to incomplete debridement and obturation of the root canal space, missed canals, fractured instruments, perforated roots and non-healing *periapical*infections [4-6]. Studies that examined morphology of mandibular first and second molars have revealed variation in the number of roots and the root canals, among Sudanese, Ugandan, Mongoloid and Caucasian ethnic groups [1, 7-9]. Average tooth length of mandibular molars has been reported to show some divergence among different ethnic groups with the majority showing a lack of sex difference among populations with similar ethnicity [10]. A few of these studies that noted sex differences in tooth length mentioned the effect of the Y-chromosome on tooth development, stating sexual dimorphism in shape and size as being the reason for the observed difference [11].

Canal configuration types commonly reported in the mesial root of two rooted teeth were type II configurations in about 40% (two canals which join to exit as one apical foramen), and type IV configuration in 45% (two canal exits as two foramen). In the distal root (70-100%) showed type I configuration (one canal exits in one foramen) [9, 12, 13]. In the three-rooted molars, 80% of the main distal roots and 100% of the disto-lingual roots had type I canals, while the majority (66.7%) of the root canals in the mesial root were type IV [12]. Other important morphological variations which may obscure clinical detection and contribute to the failure of root canal treatment are associated with the location of the apical foramen and presence of an apical delta. The apical foramen may be located centrally or laterally (mesial, distal, labial or lingual). In the few studies of Caucasian populations, the prevalence of an apical delta, an area of many small canals through which the blood and nerve supply enter and leave the pulp cavity, ranged from 2% to 6%, with a high prevalence of 10% in the mesial root of the mandibular first molar [7, 14]. Literature searches have revealed that very few studies on root canal morphology have been done in Africa [8, 9, 15] and currently, there is no retrieval data on the frequency of root canal morphology variations from Tanzania. Furthermore, Tanzanian dental practitioners have been relying on the guidelines formulated by *quality assurance guideline for endodontics as stipulated by European Society of Endodontology* [16] based on data collected from different ethnic populations. This may increase the risk of treatment failure particularly in Tanzanian populations with teeth that may have different frequencies of anatomical variations. In order to increase the success rate of root canal treatment in the Tanzanian population, it is imperative to know the root canal morphology and the common variations. The aim of this study, therefore, is to determine the typical root canal morphology of mandibular molar teeth and identify any variations and their frequencies that may exist between native Tanzanians and that reported in the literature.

## Methods

**Teeth collection site**: This article is part of a bigger descriptive cross sectional in-vitro study of extracted human adult permanent teeth. A total of 379 teeth extracted as part of treatment for patients attending public dental clinics were collected during the data collection period of March to August 2009. This was sample for convenience on clinics representing the three districts within Dar es Salaam, (Mwananyamala, Temeke and Magomeni health centers) and a national referral hospital (Muhimbili national hospital). The inclusion criteria was permanent adult teeth which had no visible evidence of root fracture. Deciduous and young permanent teeth with open apices or teeth with a fractured root and teeth that were grossly carious and involving all the cusps were excluded. At the dental clinics, the extracted teeth were placed in labeled containers with 10% formalin solution (indicating the type of tooth and sex of the patient). This article reports on the analysis of 231 molar teeth extracted from adult patients (18 years and above) Tanzanian natives of African descent.

**Teeth processing**: The teeth were sent for processing to the Department of Restorative Dentistry of the Muhimbili University of Health and Allied Sciences (MUHAS). Access cavities were prepared using a high-speed handpiece with a variety of diamond burs (Mani Inc, Tochigi, Japan). This was followed by an exploration of the pulpal floor using an endodontic explorer to locate the root canal orifices. Teeth with prepared access cavities were then immersed in 5% solution of sodium hypochlorite (NCG Chemical Industries LTD, Dar es Salaam, Tanzania) for 48 hrs to dissolve organic tissues and pulp remnants. The solution was changed after 24 hours so as to ensure a consistent strength of the solution. After the 48 hrs, the teeth were thoroughly rinsed in running tap water for two hours after which the teeth were dried with paper napkins and placed in dry labeled containers ready for transportation to the pathology laboratory of the School of Medicine, MUHAS. Methylene blue ink (CI 52015, Pacegrove, UK. Batch no.M33776/1) was then injected into the pulp chamber using a hypodermic needle gauge 27 (Neomedic LTD, UK) and the teeth were then immersed in methylene blue alkaline for 5 days followed by washing the teeth in running tap water for four hours. The teeth were then rinsed with 5% nitric acid prior to being immersed in 5% nitric acid (Scharlau chemic, South Africa.) for 5 to 10 days. Every 24 hours the acid was changed to ensure consistency. Demineralization was assessed by checking the softness of the tooth on the application of finger pressure and complete demineralization was confirmed by radiographs of randomly selected teeth from each container. Dehydration of the decalcified teeth was done sequentially by using 50%, 70%, 95% and absolute ethyl alcohol Rochelle Chemicals, South Africa) consecutively for 30 minutes. Clearing was done by immersing the dehydrated teeth in 98% methyl salicylate May and Baker LTD, Dagenham, England) for 24 hours.

**Figure 1:**
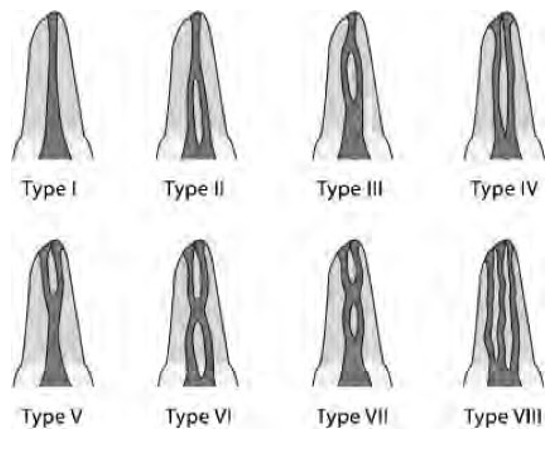
Diagrammatic representation of Vertucci’s classification of canal configuration type

**Teeth examination and recording**: Teeth were examined using a magnifying glass (10 X) for: the number of root canals, canal configuration type using Vertucci's classification (Figure 1), presence of an apical delta, location of the apical foramen, as well as tooth length which was measured from the highest cusp tips to the apex of longest root of the molar using a vernier caliper (HELIOS-PREISSER GmbH, Gammertingen, Germany).

**Data analysis**: The data collected was entered into a computer using the SPSS version 20 for statistical analysis. The unit of analysis was the tooth whereby, frequency distribution of: 1) number of roots, 2) length of tooth, 3) number of canals in a root, 4) location of apical foramen, 5) presence of an apical delta and 6) canal configuration type using Vertucci's classification, was recorded. Mean tooth length with the standard deviation was computed and chi-square tests were used to determine a statistically significant association by sex.

**Ethical consideration**: Ethical clearance and permission to handle human materials were sought from the research and publication committee of Muhimbili University of Health and Allied Sciences (MUHAS) and permission to conduct the study was obtained from the district hospital administration. Verbal informed consent was obtained from all subjects whose teeth were used in this study. There was no direct contact between the patients and the researcher.

## Results

**Figure 2:**
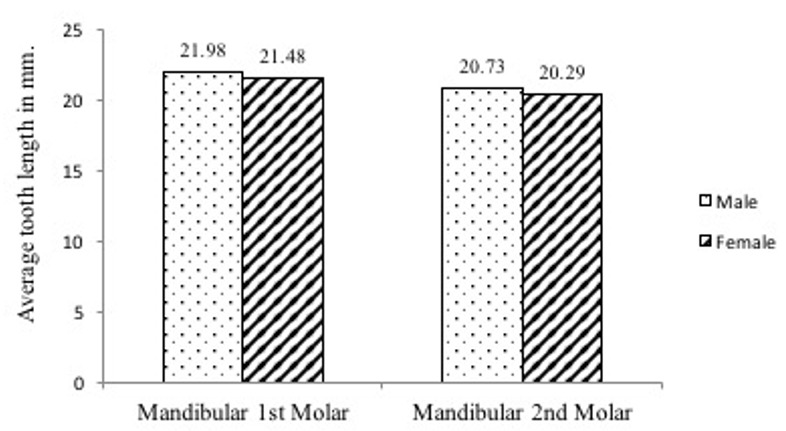
Average tooth length distribution by sex and tooth type

A total of two hundred and thirty-one (231) extracted mandibular molar teeth were used in this study. There was a total of one hundred and forty-six (146) lower first molars and eighty-five (85) lower second molars. The average tooth length for the mandibular first and second molars was 21.67 (SD1.8) mm and 20.51 (SD1.7) mm, respectively. Figure 2 shows that males (N=55) have slightly longer teeth than females (N=91) in all teeth types. This difference was not statistically significant with p-values of 0.595 and 0.630 for first and second mandibular molars, respectively. All of the first and second molars were two-rooted, having one mesial and one distal root. The mesial roots of all the first and second molars had two root canals. The majority of the distal roots of 1^st^ molars (59.6%, N=87) and 2^nd^ molars (54.1%, N=46) had single and double canals, respectively (Table 1). Table 2 shows that majority of mesial (65.1%) and distal (59.6%) roots of mandibular 1^st^ molars have canal configuration type II (i.e. two canals arising from the pulp chamber joining to form one apical foramen) and type I (one canal exiting in one foramen), respectively. Although in smaller percentages, a similar trend is portrayed in the 2^nd^ molar, with the majority of mesial roots having type II (51.8%) followed by type IV (two canals from the pulp chamber with two apical foramen) in 47.1%; while the distal root mainly exhibiting type I (45.9%) configuration. A higher percentage of the distal roots of first (59.6%) and second (45.9%) mandibular molars had canal configuration type I (Table 2). The majority of the roots which had two root canals (i.e. 26.7% and 31.8% for first and second molars, respectively) had configuration type II and fewer teeth had canal configuration type IV (13.7% and 22.4% for first and second molars, respectively). Only one mesial root of mandibular 1^st^ molar (0.7%) and one mesial root of mandibular 2^nd^ molar (1.2%) had canal configuration III. The apical foramen of all teeth analyzed were found to be located in the central position. Furthermore, an apical delta was evident in only one (1) tooth, which was in the distal root of a mandibular second molar.

**Table 1:** Percentage distribution in mandibular 1^st^ and 2^nd^ molars according to the number of canals in their distal root (N= 231)

Tooth type	One distal canal	Two distal canals	Total
Mandibular 1^st^molars	87 (59.6%)	59 (40.4%)	146 (100%)
Mandibular 2^nd^molars	39 (45.9%)	46 (54.1%)	85 (100%)

**Table 2:** Root canal configuration type of mandibular 1^st^ and 2^nd^ molars, N(%)

1^st^ molar^*^	Type I	Type II	Type III	Type IV	Type V	Type VI	Type VII	Type VIII
Mesial	-	95 (65.1%)	1 (0.7%)	50 (34%)	-	-	-	-
Distal	87 (59.6%)	39 (26.7%)	-	20 (13.7%)	-	-	-	-
2^nd^ molar**								
Mesial	-	44 (51.8%)	1 (1.2%)	40 (47.1)	-	-	-	-
Distal	39 (45.9%)	27 (31.8%)	-	19 (22.4%)	-	-	-	-

* 1^st^ mandibular molar N=146

** 2^nd^ mandibular molar N = 85

## Discussion

Mandibular molars have been reported to be the most afflicted and hence the most extracted teeth in Tanzania [17, 18]. Although the reason for extraction was not known in this study, mandibular molars were featured as the most extracted tooth type in various dental clinics that provided the specimens. This could probably be due to the fact that the commonly reported caries lesions in a native Tanzanian population as being the pit and fissure type. Similar to countries with established dental restorative care, mandibular molars are reported to be the most restored teeth in the adult dentition [5].

**Tooth length**: Tooth length determination during treatment is aided well when a clinician has an underlying presumption about the length of tooth. The average tooth length reported in this study differed from other studies. The difference could be related to ethnicity, or to the use of different measuring devices, such as radiographic measurement and electronic measurement using apex locators or different techniques. In the present study, length measurement was performed on extracted teeth, a method reported to be quite accurate [2]. Specifically, average tooth length of the mandibular first molars in this study is similar to what has been reported by Grossman [7]. The mandibular second molar having an average length of 20.51 mm is lower in comparison to that found by Grossman [7] who reported an average length of 22.4 mm. In line with what was observed in the current study, the few studies that have examined the sex differences in tooth length, a study by Alam and co-workers [10] among the Bangladeshi population, who reported no statistically significant sex difference in tooth length.

**Number of roots**: All mandibular 1^st^ and 2^nd^ molars in this study had two roots, which is reported to be a common finding in these teeth. There are documented instances of mandibular first molars exhibiting an additional lingual root referred to as radix entomolaris. The occurrence of these three rooted first molars is less than 3% in Africans, 4.2% in Caucasians, and 5% in Asians but higher than 5% in Mongoloid populations [1]. The later finding (radix entemolaris) did not appear in this study, possibly due to utilization of extracted teeth which had no fractures, which might be a reason. The current finding of having two roots though, is similar to what has been reported in a Ugandan population [9] as well as among Caucasians [14]. The latter was unlike what has been reported to occasionally occur on 1^st^ mandibular molars of Sri Lankan (95.8%), Taiwan Chinese (77%) and Indian (94.6%) adult populations [19-21]; as well, in Sudan where 97% and 78% of the 1^st^ and 2^nd^ molars, respectively, exhibited two roots [8].

**Number of canals of each root of mandibular molars**: The finding from this study that all mesial roots in all mandibular molars exhibited two root canals, differs slightly from other studies that reported two canals in the mesial root of 1^st^ mandibular molars, among 86% of Sudanese, [8], 96% of Iranian [22], 96% of Kuwait [23], 96% of Taiwan Chinese [19] and 96.3% of Kenyan [15] populations. This variation eighty-five in the mesial root of 2^nd^ mandibular molars, in 83% of Sudanese population [8]. The occurrence of two root canals in distal roots of lower first molar in this study of 40% (Table 1), is somewhat similar to what has been reported in Taiwan-Chinese (46%), Kuwait (49%), Iranian (34.4%) and Kenyan (43%) population [15, 19, 22, 23]. However, this finding is lower than that reported from other studies where two root canals in distal roots were reported to have a higher incidence, occurring in 59% of Sudanese [8] and 57.7% of Saudi Arabian [24] populations. Furthermore, the finding from this study that more than half (54%) of the distal roots of the mandibular second molars exhibiting two root canals is higher than that reported in studies by Ahmed and colleagues [8] of a Sudanese and Rahimi [13] of Iranian population, who reported a higher occurrence of a single root canal instead (that is 69% and 77.5%, respectively). The difference in the occurrence of root canals in different roots in the current study versus previous studies could be explained by a difference in the genetic make-up.

**Root canal configuration types in mandibular molars**: **Table 2** similar to results in this study, the majority of distal roots of Burmese, Thai and Turkish population have been reported to have a type I configuration [12, 25, 26]. Whilst the distal roots with two canals were, as well, reported to have configuration type II and IV [8, 22, 23]. Despite the favorable canal morphological features of Tanzanian adult's mandibular molars, the configurations type III reported in a few could be the source of root canal treatment failure, since they may be left un-instrumented and un-filled and hence a source of infection.

**Apical delta**: The low number of mandibular molars with an apical delta observed in this study is similar to what has been reported among Brazilians whereby 0.95% of treated teeth mandibular molars exhibited apical deltas [27]. In the Jordanian population [2], none of the examined mandibular molar teeth were found to have an apical delta. This is contrary to the report by Vertucci, [14] showing among Caucasians, a prevalence of an apical delta ranging from 6% on mesial root of second molar to 14% on the distal root of first mandibular molars. However, the finding in this study and others could be due to the methodology employed since it did not involve the application of apical suction pressure to assist in dye penetration, which may have resulted in less penetration of the dye in these areas. Although the presence of apical deltas was lower when compared to other studies, clinicians should always look for possibilities of their occurrence. Proper sealing of apical deltas usually brings about reduced infection in root canal treated teeth.

**Location of apical foramen**: The central location of apical foramen among a sample of Tanzanian adult teeth in the current study differed from Sert and co-worker's [3] study among the Turkish population that reported the apical foramen of lower molar teeth to be mainly located laterally. Generalizations from these findings should be taken with caution since the teeth were collected mainly from the dental clinics in Dar es Salaam. This in-vitro study of root canal morphology of mandibular permanent teeth was conducted after obtaining extracted teeth from different dental clinics within Dar es Salaam. Measures were taken to control collection of each tooth type by sex. This was done by ensuring that the extracted tooth was immediately placed into the container with an appropriate label after each extraction procedure. Different techniques have been used to determine the root canal morphology of teeth. These include the use of radiographs, sectioning, decalcifying and clearing and computer-aided techniques [28-31]. The decalcifying and clearing technique used in this study, though not comparable with more recent sensitive techniques, is inexpensive, simple to perform in a Tanzanian setting and does not require the use of expensive machines. Furthermore, this technique also gives a three-dimensional view of the tooth under study. Also, by using this technique, instrument manipulation of the pulp system is not necessary so the original form of the pulp system is maintained. Notwithstanding these limitations, these findings form a baseline database on tooth morphology variations in mandibular molars of Tanzanian native adult population. Further research is recommended to study root morphology that will utilize a larger sample from different parts of the country to obtain a more representative sample, which would increase the chance of identifying rare morphological variations, by utilizing modern techniques such as Micro-Computed Tomography and Cone-Beam Computed Tomography which are non-destructive methods.

## Conclusion

Variation in the morphology in a sample of Tanzanian adult's mandibular molars is common, with the canal configuration type I, II and IV, that require a clinician's attention during treatment. The occurrence of apical foramen has been found to be favorably located with infrequent occurrence of apical deltas. Clinicians performing root canal treatment in Tanzania should consider anatomical variations in their patients since they occur frequently so as to get the most of the patient root canal treatment outcomes.

### What is known about this topic

- There is variation in root and canal morphology among adults of different ethnicity;
- Mandibular molars do encounter differing root canal configuration when classified according to Vertucci.

### What this study adds

- First study on root canal morphology of mandibular molars of native Tanzanian adults;
- Provides baseline information that there are some variations in root canal morphology of mandibular molars among native Tanzanian adults.

## Competing interests

The authors declare no competing interest.
